# Mechanism of PARP inhibitor resistance and potential overcoming strategies

**DOI:** 10.1016/j.gendis.2023.02.014

**Published:** 2023-03-24

**Authors:** Xiaoyu Fu, Ping Li, Qi Zhou, Ruyuan He, Guannan Wang, Shiya Zhu, Amir Bagheri, Gary Kupfer, Huadong Pei, Juanjuan Li

**Affiliations:** a Department of Oncology, Georgetown Lombardi Comprehensive Cancer Center, Georgetown University Medical Center, Washington, DC 20057, USA; b Department of Breast and Thyroid Surgery, Renmin Hospital of Wuhan University, Wuhan, Hubei 430060, China; c Cancer Center, Renmin Hospital of Wuhan University, Wuhan, Hubei 430060, China; d Department of Thoracic Surgery, Renmin Hospital of Wuhan University, Wuhan, Hubei 430060, China

**Keywords:** Drug resistance, Homologous recombination, PARP, PARP inhibitor, Poly (ADP-ribose) polymerase

## Abstract

PARP inhibitors (PARPi) are a kind of cancer therapy that targets poly (ADP-ribose) polymerase. PARPi is the first clinically approved drug to exert synthetic lethality by obstructing the DNA single-strand break repair process. Despite the significant therapeutic effect in patients with homologous recombination (HR) repair deficiency, innate and acquired resistance to PARPi is a main challenge in the clinic. In this review, we mainly discussed the underlying mechanisms of PARPi resistance and summarized the promising solutions to overcome PARPi resistance, aiming at extending PARPi application and improving patient outcomes.

## Introduction

Poly (ADP-ribose) polymerase (PARP) is a multifunctional post-translational modification enzyme, which participates in various biological processes.^1^ Among all the 17 members of the poly (ADP-ribose) polymerases (PARPs) family, PARP1 is a particularly important protein for DNA damage repair and genome stability, which catalyzes the covalent attachment of PAR polymers on itself and other specific proteins, including histones, DNA repair proteins, and chromatin modulators using NAD^+^ as a donor of ADP-ribose units.^2^, ^3^, ^4^ PARP1 contains several domains: N-terminal zinc finger motif, BRCT domain, WGR domain, and C-terminal catalytic (CAT) domain.^5^ PARP1 plays an essential role in repairing single-strand breaks (SSBs).^6^ Since SSBs are an intermediate of base-excision repair (BER),^7^ PARP1 is also required for BER.^8^ Once DNA damage occurs, PARP1 is activated and initiates the auto-PARylation, which further activates PARP1 and enables the PARylation of histones and other DNA damage-associated proteins. Eventually, this auto- and hetero-modification recruit the downstream DNA repair proteins, such as XRCC1, to the DNA damage site, promoting the effective repair of DNA.^9^^,^^10^

PARP1 is also cleaved between Asp124 and Gly215 by caspases, core members of apoptosis,^11^ resulting in two specific segments: the catalytic domain (89 kD) of the carboxyl-terminal fragment and DNA-binding domain (24 kD) of the amino-terminal segment.^11^ In addition to the role of PARP1 in DNA damage repair and apoptosis, PARP1 also regulates the activity of RNA polymerase II by inhibiting the negative elongation factor (NELF) and modulating the gene transcription process.^12^

The homologous recombination (HR) repair pathway is a major pathway to repair the DNA double-strand breaks (DSBs), and HR deficiency leads to genomic instability and tumorigenesis.^13^ PARP inhibitor (PARPi), which inhibits DNA SSBs repair, exerts a synthetic lethality with tumor-specific HR deficiency and presents the anti-tumor effect. Emerging evidence supports the anti-tumor role of PARPi in both inherited cancer with BRCA mutation, and in sporadic cancer harboring HR repair deficiency.^14^ Synergistic effects were observed in the combination treatment with PARPi and platinum-based chemotherapy. Platinum can covalently crosslink DNA to cause DSBs, while PARPi can inhibit DNA single-strand repair.^15^ Therefore, patients with platinum-sensitive tumors are more likely to benefit from the therapy of PARPi. Even in patients with BRCA mutations, the efficacy of PARPi is still related to the response to platinum.^16^ Although PARPi is a promising treatment, the majority of patients will relapse due to acquired resistance. PARPi resistance is becoming a challenge, threatening the efficacy of PARPi. There is a critical need to identify the molecular mechanisms of resistance to PARPi and explore strategies to overcome the resistance.

## The mechanism of action of PARPi

**Figure 1:**
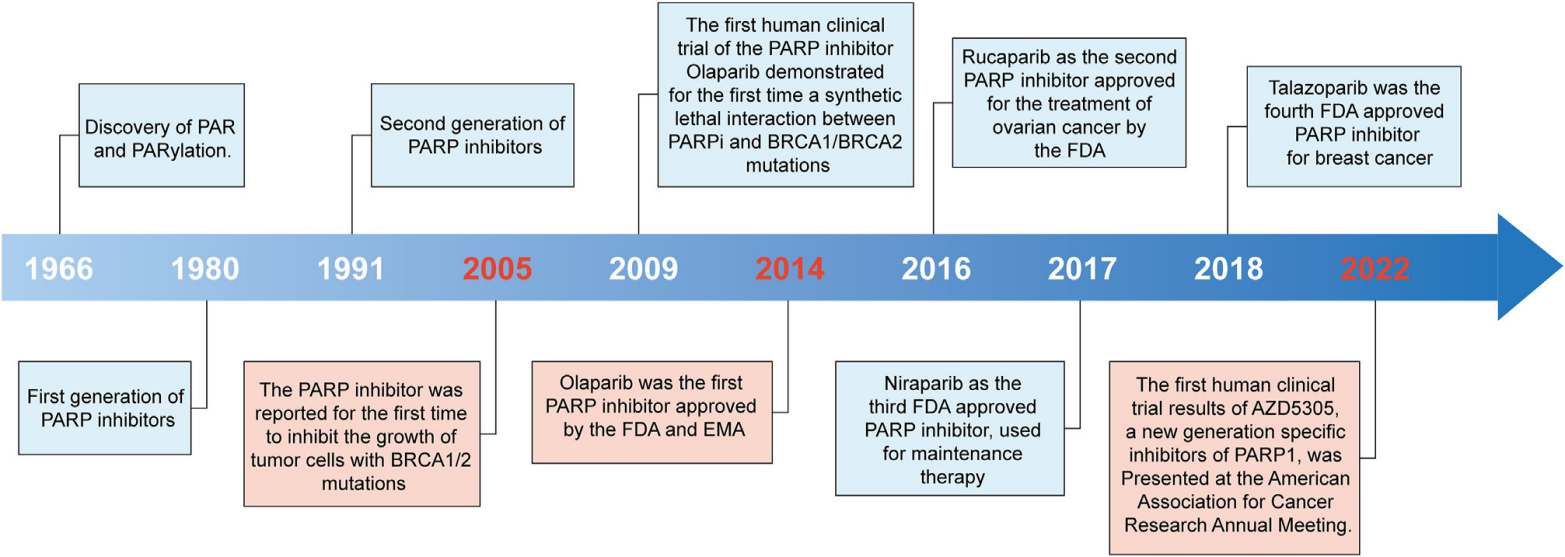
Hallmarks in the field of PARP inhibitors.

Since 2014, when olaparib (AZD2281) was granted accelerated approval by the United States Food and Drug Administration (FDA) as monotherapy in inherited BRCA-mutated ovarian cancer,^17^^,^^18^ PARP inhibitors, including olaparib, rucaparib, niraparib, and talazoparib, have received approvals by FDA to be used in various cancers based on their revolutionary results in clinical trials (Fig. 1). PARP1, the most extensively studied PARP member, will be used as an example to illustrate the mechanisms of PARPi.

### PARP1 catalytic inhibition

**Figure 2:**
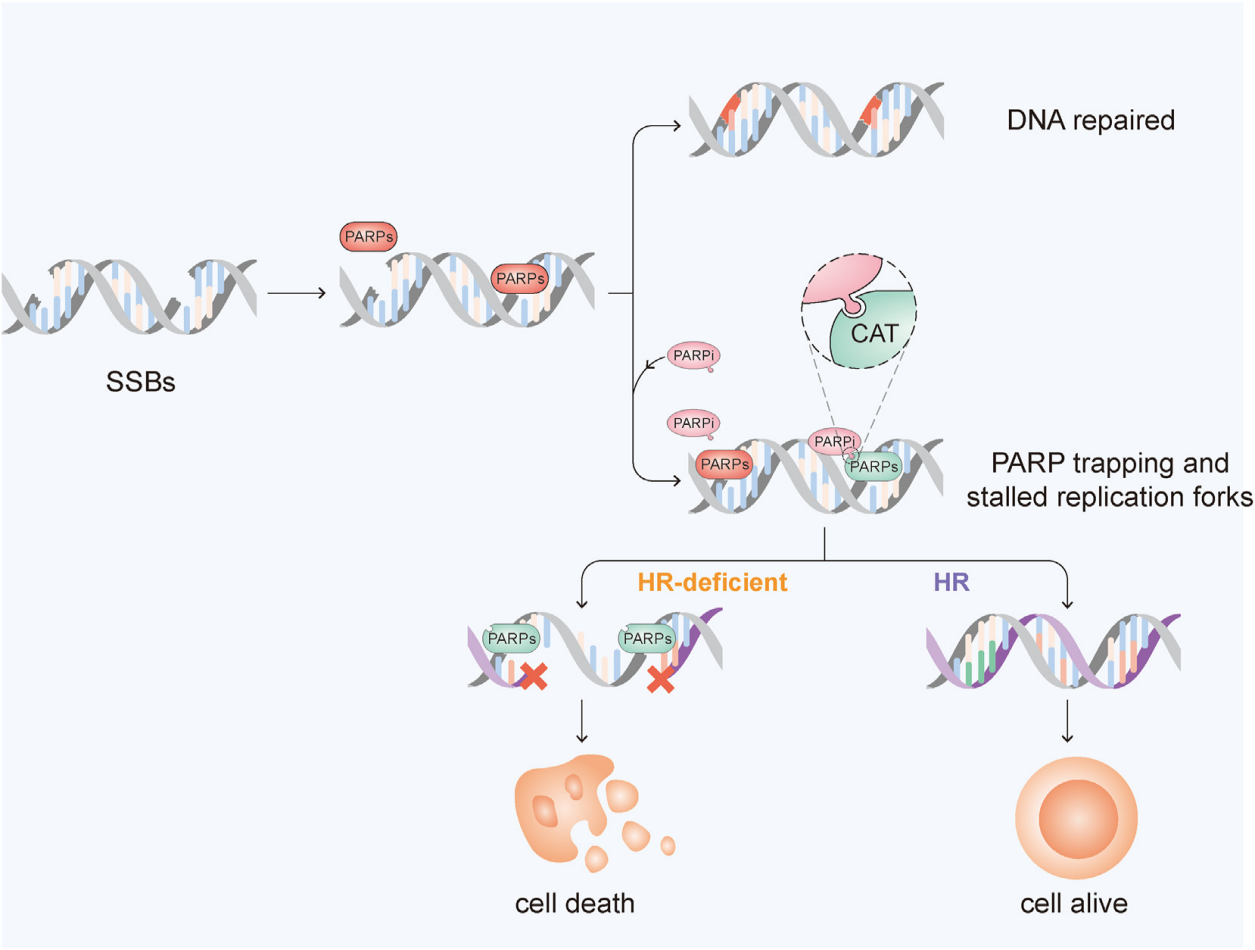
The mechanism of action of PARPi. PARPs are recruited to the sites of DNA single-strand breaks (SSBs) and rapidly activated, which initiate and maintain DNA damage repair. PARPi contains a nicotinamide moiety that binds PARPs, which blocks the synthesis of PAR chains and interferes with the repair of SSBs. PARP1 autoPARylation leads to its release from the site of DNA damage. After the competitive binding of PARPi, PARPs are allosteric and enhance the binding strength to damaged DNA. Trapped PARPs lead to the accumulation of unrepaired SSBs, which impair the proper progression of replication forks and ultimately result in the formation of DSBs that need homologous recombination (HR) repair. If the HR repair is deficient, the DNA breaks might not be repaired and ultimately lead to cell death.

PARP1 is recruited to the site of SSBs and its C-terminal catalytic (CAT) domain can be rapidly activated to hydrolyze NAD^+^, leading to the PARylation of several proteins as well as itself,^19^^,^^20^ which initiates DNA repair mechanisms (Fig. 2). This binding of NAD^+^ to PARP1 recruits DNA repair proteins to the site of DNA damage.^21^ PARP inhibitor is a nicotinamide analogue bearing the nicotinamide moiety that could compete with NAD^+^ to bind PARP1.^22^ PARPi kills cancer cells by blocking the synthesis of PAR chains and interfering with the repair of SSBs. PARPi does not affect the condensation level of undamaged DNA but blocks the reversal of condensation of damaged DNA in the presence of NAD^+^.^23^

### PARP1 trapping

PARPi also works through PARP1 trapping. In general, PARP1 autoPARylation induces itself to release from the DNA damage site.^24^ After the competitive binding of PARPi, PARP1 is allosteric, which enhances PARP1 binding to damaged DNA.^25^ The trapped PARP1 on SSBs encounters replication forks and then leads to DSBs that need to be repaired by HR.^26^ If PARPi is used on HR-deficient cells, the DNA breaks could not be repaired, which may impede replication forks and ultimately lead to cell death.^27^ Thus, trapped PARP1-DNA complexes are more cytotoxic than unrepaired SSBs caused by PARP1 inactivation. After being captured, PARP1 is SUMOylated by PIAS4 (protein inhibitor of activated STAT 4) and subsequently ubiquitylated by the Sumo-targeted E3 Ubiquitin ligase RNF4, promoting recruitment of P97 and removal of trapped PARP1 from chromatin. P97 complex inhibitors prolong PARP1 trapping and enhance PARPi-related cytotoxicity in HR-deficient tumor cells.^28^ The elevated repairability of PARP-DNA complexes is linked to acquired drug resistance.

### Replication gap

The replication gap is another key determinant of the mortality of PARPi in BRCA defective cells. BRCA1-or FANCJ-deficient cells exhibit common repair deficiencies and distinct PARPi responses, implying that the replication gap might be a key differentiating factor. The sensitivity to PARPi aligns with the extent of replication gap formation, and replication gaps in BRCA1-deficient cells are caused by Okazaki fragment processing (OFP) defects. Targeting gaps can resensitize and augment PARPi synthetic lethality.^29^ Spartan (SPRTN), a metalloprotease involved in DNA replication, is associated with the response to various PARP inhibitors. SPRTN-deficient cells were sensitive to olaparib and talazoparib since SPRTN-deficient cells showed delayed clearance of trapped PARP1 and replication fork delay when treated with talazoparib or olaparib.^30^ However, SPRTN-deficient cells were not sensitive to PARP trapper veliparib.

## Current status of PARPi drugs

Olaparib is the first PARPi validated in clinical trials, which demonstrated its efficacy and safety in several malignancies. Thus far, olaparib, niraparib, talazoparib, and rucaparib have been approved for selected patients with breast, ovarian, or pancreatic cancer.^31^^,^^32^ The timeline for the development of PARPi is shown in Figure 1, and the clinical progress of several major PARP inhibitors is summarized in Table 1.

**Table 1:** Clinical progress of several major PARP inhibitors.

Drug	Types of cancer	Approval (FDA/EMA/NMPA)	Ongoing clinical trials			
			Clinical trials	Phase	Therapy	Inclusion criteria
Olaparib	Ovarian cancer	(i) Advanced ovarian cancer with gBRCA mutations (ii) Maintenance for advanced ovarian cancer with gBRCA mutated or HRD.	NCT03737643	3	Durvalumab + bevacizumab + olaparib after durvalumab + chemotherapy + bevacizumab	Newly diagnosed advanced ovarian cancer
	Breast cancer	(i) Metastatic gBRCA-mut HER2-negative breast cancer (ii) Early gBRCA-mut HER2 negative breast cancer with high risk	NCT03150576	3	Olaparib + platinum	TNBC in the neoadjuvant setting
	Pancreatic cancer	Metastatic gBRCA-mut pancreatic adenocarcinoma				
	Prostate cancer	Metastatic castration-resistant prostate cancer with HRR gene-mutated.	NCT03732820	3	Olaparib + abiraterone + prednisone	mCRPC
Rucaparib	Ovarian cancer	Metastatic ovarian cancer with gBRCA mutations.	NCT03522246	3	Rucaparib + nivolumab	Metastatic ovarian cancer sensitive to platinum
			NCT04227522	3	Maintenance therapy	Metastatic ovarian cancer
			NCT02855944	3	Rucaparib	Metastatic ovarian cancer
	Prostate cancer	Metastatic castration-resistant prostate cancer with BRCA-mut	NCT02975934	3	Rucaparib	Metastatic castration-resistant prostate cancer with HRD
			NCT04455750	3	Rucaparib + enzalutamide	Metastatic testosterone-deprivation-resistant prostate cancer
Niraparib	Ovarian cancer	(i) Platinum-sensitive advanced ovarian cancer with HRD. (ii) Maintenance treatment in platinum-sensitive advanced ovarian cancer.	NCT05460000	3	Niraparib maintenance after chemotherapy	Advanced ovarian cancer with HRD
			NCT03705156	3	Niraparib maintenance after chemotherapy	Advanced platinum-sensitive ovarian cancer with HRD
			NCT03598270	3	Niraparib maintenance with/without atezolizumab after chemotherapy with/without atezolizumab	Advanced ovarian cancer
			NCT05009082	3	Niraparib with/without bevacizumab	Advanced ovarian cancer
	Breast cancer		NCT04915755	3	Niraparib	Early BRCA-mut HER2 negative breast cancer with and TNBC with detected ctDNA
	Prostate cancer		NCT04497844	3	Niraparib + abiraterone acetate + prednisone	Metastatic castration-sensitive prostate cancer with HRR gene-mutated
			NCT03748641	3	Niraparib + abiraterone acetate + prednisone	Metastatic prostate cancer
	Non-small cell lung cancer		NCT04475939	3	Niraparib + pembrolizumab as maintenance therapy	Stage IIIB/IIIC or IV non-small cell lung cancer sensitive to platinum and pembrolizumab
Talazoparib	Breast cancer	Advanced gBRCA mutated HER2-negative breast cancer				
	Ovarian cancer		NCT03642132	3	Avelumab + talazoparib as maintenance therapy	Newly diagnosed locally advanced or metastatic ovarian cancer
	Prostate cancer		NCT04821622	3	Talazoparib + enzalutamide	DDR-deficient mCSPC
			NCT03395197	3	Talazoparib + enzalutamide	Metastatic castration-resistant prostate cancer
Fuzuloparib (NMPA)	Ovarian cancer	advanced BRCA-mut platinum-sensitive ovarian cancer				
Veliparib	Breast cancer		NCT02163694 (has results)	3	Carboplatin + paclitaxel + veliparib	HER2-negative BRCA-associated breast cancer
	Ovarian cancer		NCT02470585	3	Veliparib + carboplatin + paclitaxel	Advanced ovarian cancer
IMP4927	Ovarian cancer		NCT04169997	3	IMP4297	Advanced ovarian cancer
Pamiparib	Ovarian cancer		NCT03519230	3	Pamiparib	Platinum-sensitive recurrent ovarian cancer
AZD5305	Prostate Cancer		NCT05367440	I/IIa	AZD5305 + new hormonal agents	Metastatic prostate cancer
	solid tumors		NCT04644068	I/IIa	AZD5305 with/without anti-cancer agents	Advanced solid malignancies

Note: A platinum-sensitive tumor is defined as a tumor that does not recur within 6 months or more following platinum-based therapy. BRCA, breast cancer gene; DDR, DNA damage response; EMA, European Medicines Agency; FDA, Food and Drug Administration; NMPA, National Medical Products Administration; gBRCA, germline BRCA; HER2, human epidermal growth factor receptor 2; HRD, homologous recombination deficiency; HRR, homologous recombination repair; mCSPC, metastatic castration-resistant prostate cancer.

### Olaparib

In 2009, the first clinical trial of the PARPi demonstrated a synthetic lethal for olaparib in breast cancer with BRCA1/BRCA2 deficiency.^33^ As more clinical trials have demonstrated its efficacy and safety, olaparib has been approved for pancreatic cancer, breast, and ovarian with BRCA1/2 mutations. A recent 5-year follow-up data from a randomized, double-blinded, phase III trial (SOLO1/GOG 3004) showed that the benefits of 2 years of olaparib maintenance continued until the end of treatment with newly diagnosed advanced ovarian cancer with BRCA mutations. Median progression-free survival (PFS) is prolonged to 4.5 years.^34^ SOLO2 trial also demonstrated that olaparib significantly improved overall survival (OS) and PFS as maintenance therapy in platinum-sensitive recurrent ovarian cancer (PROC) patients with BRCA mutations. However, post-hoc analyses suggested that patients who progressed after olaparib had less benefit from subsequent platinum-based chemotherapy than those who did not receive PARPi. Time to second progression was significantly longer in the placebo group than that in the olaparib group (12.1 months *vs*. 6.9 months).^35^ In clinical trials of metastatic castration-resistant prostate cancer (mCRPC), they revealed the synergy effect of olaparib combined with cabazitaxel in mCRPC patients who relapsed after docetaxel and androgen receptor axis targeted therapy.^36^ Furthermore, olaparib showed a longer PFS and a higher response rate.^36^^,^^37^ The exploratory analysis of the TOPARP-B trial showed that prostate cancer patients with homozygous loss of BRCA2, PALB2, and ATM derived more benefit from the addition of olaparib.^38^ For the mCRPC patients with DNA-repair defects who failed prior hormonal therapy, olaparib monotherapy also showed higher response rates and longer PFS compared to enzalutamide or abiraterone.^39^^,^^40^ A recent study suggests that olaparib maintenance therapy could bring survival benefits to metastatic pancreatic cancer patients with germline BRCA1/2 (gBRCA1/2) mutations.^41^

### Rucaparib

Rucaparib maintenance is a safe and effective therapy for platinum-sensitive advanced pancreatic cancer with BRCA1/2 or PALB2 pathogenic variant.^42^ ARIEL2, a single-arm phase II trial, revealed the efficacy and safety of rucaparib in relapsed, high-grade ovarian cancer.^43^ It also proved that RAD51C and RAD51D mutations, as well as hypermethylation of BRCA1 promotors, were associated with better responses to rucaparib. The loss of BRCA1 methylation may be the main mechanism of cross-resistance between platinum and PARPi.^43^

### Niraparib

*De novo* advanced ovarian cancer patients who received niraparib had significantly longer PFS regardless of the presence of HR defects.^44^ Niraparib is a useful option for maintenance therapy for relapse ovarian cancer sensitive to platinum.^45^ Additionally, niraparib is shown to significantly prolong PFS with manageable toxicity for patients with platinum-sensitive, extensive-stage small cell lung cancer (ES-SCLC) when used for maintenance therapy.^46^

### Talazoparib

Talazoparib got approval for the treatment of HER2-negative metastatic breast cancer with gBRCA1/2 mutations due to its efficacy and safety.^47^ Talazoparib showed durable anti-tumor activity in patients with heavily pretreated advanced anti-castration prostate cancer altered by the DNA damage response (DDR)-HR genes.^48^

### Fluzoparib

In a single-arm, phase II study, fluzoparib showed a good anti-tumor activity with an acceptable safety profile for patients with platinum-sensitive recurrent ovarian cancer with gBRCA1/2 mutations.^49^ A phase III FZOCUS-2 trial demonstrated a significant improvement in PFS with the utility of fluzoparib for maintenance therapy in platinum-sensitive, relapsed ovarian cancer patients, regardless of BRCA1/2 status.^50^

### AZD5305

Studies have shown that the main synthetic lethal effect together with BRCA mutation is PARP1, suggesting that the inhibition effect on PARP2 may not be necessary to play a therapeutic role.^51^^,^^52^ Some PARP inhibitors failed in combination with other chemotherapies mainly owing to overlapping hematotoxicity, which is caused by its effects on PARP2 and other PARPs. Thus, finding a highly selective PARP1 inhibitor with reduced toxicity caused by cross-inhibition of PARP2 has become one of the important research directions for scientists. In 2015, Nerviano Medical Sciences first reported an effective, oral, and highly selective PARP1 inhibitor, NMS-P118, with good ADME (absorption, distribution, metabolism, and excretion).^53^ The pace of research has never stopped. AZD5305, a new generation of PARP1 specific inhibitor, is also a PARP1-DNA trapper, which showed good efficacy *in vivo* using the BRCA mutant HBC-17 patient-derived tumor xenograft (PDX) model.^54^ Giuditta et al showed that AZD5305 was 500-fold more selective for PARP1 over PARP2. Surprisingly, AZD5305 only worked on defective cells other than normal cells. This preclinical study strongly supports the hypothesis that only inhibiting PARP1 might reduce adverse events without compromising with therapeutical efficacy.^55^ The ongoing phase I/IIa PETRA trial (NCT04644068) has enrolled 61 patients with advanced HER2-negative breast, ovarian, prostate, or pancreatic cancer who had germline or somatic BRCA1/2, PALB2, or RAD51C/D loss-of-function mutations. According to the preliminary dose-limiting toxicity (DLT) assessment, AZD5305 showed promising clinical activity and safety in those patients.^56^ Its therapeutic effect in clinical patients and more specific indications for patient selection need further research in the near future.

## Mechanism of PARPi resistance

**Figure 3:**
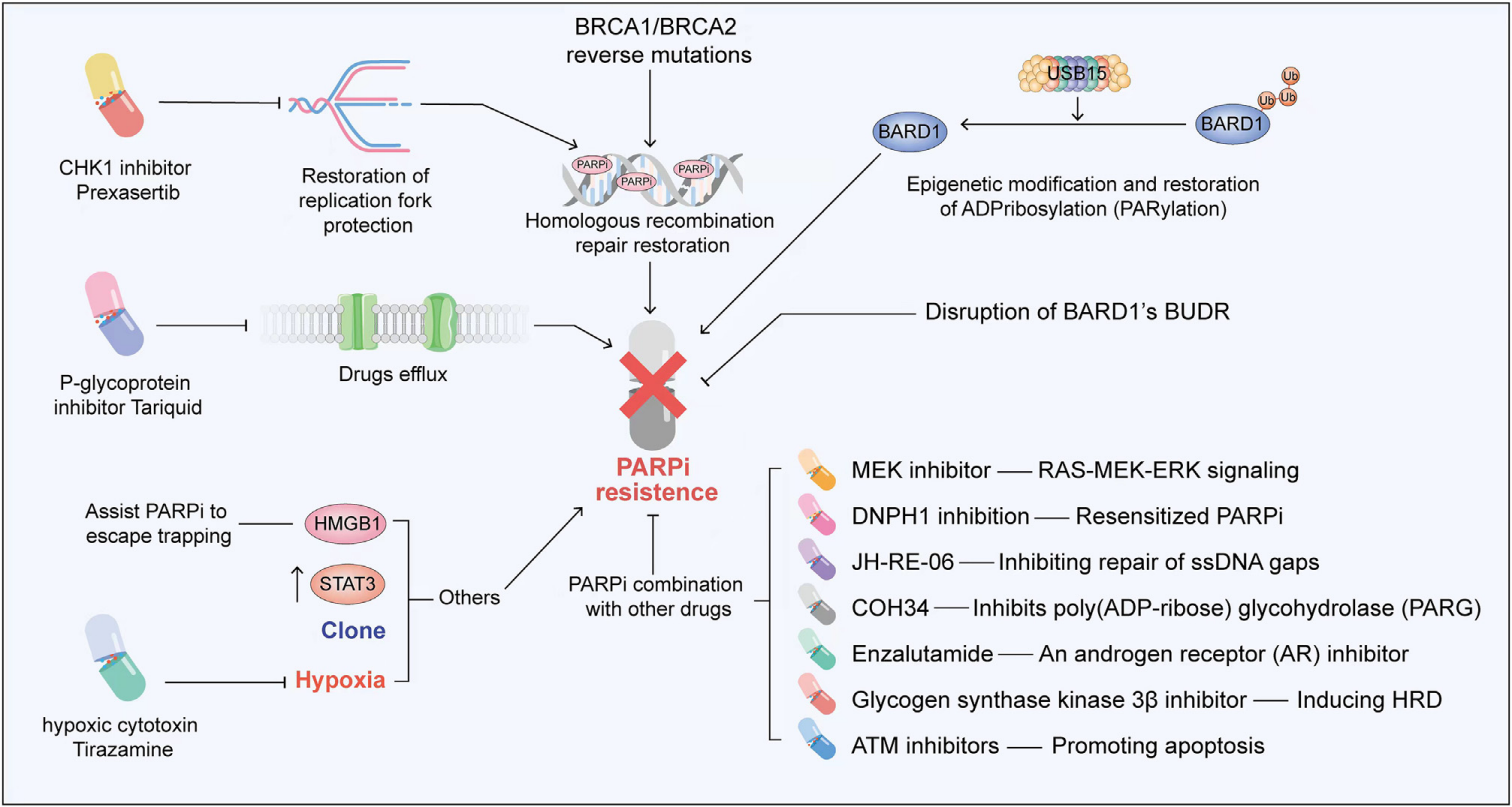
Multiple mechanisms and potential solutions of PARPi resistance. The mechanisms of PARPi resistance are complex and mainly have the following ways: (i) the increase of drug efflux; (ii) genomic reversal of BRCA1/2; (iii) restoration of replication fork protection; (iv) any strengthening of the homologous recombination repair process; (v) epigenetic modifications; and (vi) other mechanisms, such as high mobility group box 3 (HMGB3), STAT3, clonal selection, and hypoxia. This figure illustrates the main mechanisms of PARPi resistance and the combination strategies that potentially overcome PARPi resistance.

Although PARPi could bring durable survival benefits for selected patients in clinical trials and in the real world, innate and acquired resistance to PARPi has dampened the initial enthusiasm. The underlying mechanisms by which tumor cells evade therapeutic intervention and acquire drug resistance are very complex (Fig. 3). Moreover, the drug resistance mechanisms in each tumor may be the product of several drug resistance mechanisms rather than a single independent mechanism.^57^ Summaries of the potential drug resistance mechanism studies of different PARP inhibitors are listed in Table 2.

**Table 2:** The relevant drug resistance mechanism studies of different PARPi.

Drug	Mechanism of PARPi resistance	Solution	Reference
Olaparib	Enhancement of drug efflux	p-glycoprotein inhibitor	Rottenberg, 2008^58^
	BRCA reversal mutation	Inhibition of DNA end-joining repair pathways	Tobalina, 2021^65^
	Homologous recombination repair restoration	–	Mirman, 2022^71^
	High C/EBPβ expression induces restoration of HR capacity	Targeting C/EBPβ	Tan, 2021^75^
	Restoration of replication fork stability	–	Taglialatela, 2017^77^
	Promotes degradation of stalled replication forks	–	Rondinelli, 2017^78^
	The overexpression of High-mobility group box 3 (HMGB3) increased the insensitivity to PARPi,	Targeted inhibition of HMGB3	Ma, 2022^89^
	Activates STAT3	–	Martincuks, 2021^93^
	Clonal evolution	–	Anniina, 2021^94^
	Hypoxia	Eliminating hypoxic tumor cells	Mehibel, 2021^97^
Rucaparib	BRCA reversal mutation	Improve the detection of mutations	Lin, 2019^67^
	Promotes degradation of stalled replication forks	–	Rondinelli, 2017^78^
	Acquired RAD51C promoter methylation loss	–	Ksenija, 2021^82^
	Methylation of BRCA1 copies	quantitative BRCA1 methylation analysis	Kondrashova, 2018^82^
Niraparib	Acquired RAD51C promoter methylation loss	–	Ksenija, 2021^83^
	Clonal evolution	–	Anniina, 2021^94^
Talazoparib	Homologous recombination repair restoration	–	Waks, 2020^65^
	Clonal evolution	–	Anniina, 2021^94^
	Hypoxia	Eliminating hypoxic tumor cells	Mehibel, 2021^97^

### Drug efflux

Since PARPi needs to enter tumor cells to kill them, an increase in drug efflux is inevitable, affecting the therapeutic effect and eventually leading to drug resistance. The multidrug resistance protein (MDR1) encoded by ATP binding cassette subfamily B member 1 (ABCB1), is involved in the efflux of chemotherapeutic drugs and affects their effectiveness and accumulation in the brain and other tissues. Sven et al found that long-term treatment with olaparib leads to the development of drug resistance due to the up-regulation of ABCB1 encoding p-glycoprotein efflux pumps.^58^ This resistance to olaparib can be reversed by the addition of p-glycoprotein inhibitor tariquid.^58^ Therefore, inhibition of ABCB1 may have important clinical implications for patients who are resistant to PARPi.

### BRCA1/BRCA2 reverse mutations

Genomic reversal of BRCA1/2 is one of the common molecular mechanisms of PARPi resistance. Patients with defective DNA repair may recover BRCA1 or BRCA2 function through somatic reversion mutation, leading to resistance to PARPi. In several retrospective analyses of next-generation sequencing (NGS) data from 23,375 patients across various common cancer types, the prevalence of multiple pathogenic/likely pathogenic (P/LP) germline mutations in homologous recombination repair (HRR) genes was analyzed.^59^, ^60^, ^61^ The reversion mutations were detected in BRCA1 (3.8%), BRCA2 (3.5%), and PALB2 (2.0%) after platinum-based chemotherapy and/or PARPi resistance.^61^ The incidence of mutations that restore BRCA1/2 function in ovarian cancer ranges from 0% to 21%, and as high as 40% to 50% in breast cancer.^62^, ^63^, ^64^ A pooled analysis of several studies on reversion mutations revealed mutagenic end-joining DNA repair pathways, particularly those affecting BRCA2, play a key role in the generation of reversion, as indicated by a significant accumulation of microhomology in deletions surrounding DNA sequences leading to reversion events.^65^ The circulating cell-free DNA (cfDNA) sequencing analysis can help identify BRCA1/2 reversal mutations in breast and ovarian cancer patients.^63^^,^^66^ Lin et al found that in the patients treated with rucaparib, those without BRCA reversal mutations detected in pretreated cfDNA had significantly longer PFS than those with reversal mutations. BRCA reversed mutations detected in cfDNA of platinum-resistant high-grade ovarian carcinoma (HGOC) are associated with the reduced survival benefit of rucaparib therapy.^67^ Therefore, developing diagnostics for BRCA1/2 reversion mutations could pave the way for overcoming PARPi resistance.

### Homologous recombination repair restoration

Any strengthening of the HRR process may give rise to the occurrence of PARPi resistance. 53BP1 is thought to control the fidelity and the choice of DSB repair pathway.^68^ Waks et al found that in patients with gBRCA1 mutations, TP53BP1 loss or MRE11A amplification had genomic changes that restored HR by increasing DNA terminal excision.^64^ Shieldin is formed by C20orf196 (also known as SHLD1), FAM35A (SHLD2), CTC-534A2.2 (SHLD3), and REV7, as a downstream effector of 53BP1/RIF1/MAD2L2.^69^ Those could promote DSBs terminal connection by limiting DSBs resection.^70^ Recent studies found that the role of 53BP1 and Shieldin proteins in DSB depends on CST–Polα–primase fill-in synthesis and, to some extent, determines the effectiveness of PARPi on BRCA1-deficient cells.^71^ HR is counteracted by antagonizing BRCA2/RAD51 loading into BRCA1-deficient cells.^72^^,^^73^ Additionally, He et al identified DYNLL1 (dynein light chain 1 protein) as a DNA terminal excision inhibitor that could directly bind to MRE11 and reduce DNA terminal excision activity *in vitro*. The loss of DYNLL1 enables BRCA1 mutant cells to undergo DNA terminal excision and resume HR. Therefore, the loss and decreased expression of DYNLL1 leads to resistance to platinum drugs and PARPi.^74^ CCAAT/enhancer binding protein β (C/EBPβ) is an essential transcription factor of the HR pathway. Multiple HR genes (BRCA1, RAD51, BRIT1, and BRIP1) are targeted and up-regulated by C/EBPβ to induce the recovery of HR ability and mediate acquired resistance to PARPi. Deletion of C/EBPβ may be an approach to overcome resistance to PARPi.^75^

### Restoration of replication fork protection

Acquired resistance to PARPi or platinum is in connection with replication fork protection. Chaudhuri et al found that the deletion of the MLL3/4 complex protein PTIP is a key factor to protect BRCA1/2 deficient cells from DNA damage. PTIP deficiency not only restores HR activity at DSBs but also inhibits the recruitment of MRE11 nuclease to the stopped replication fork, which showed in turn the ability to protect the newly formed DNA strand from extensive degradation.^76^ In BRCA2-deficient tumors without BRCA2 reversion mutations, the acquisition of resistance to PARPi is associated with replication fork protection. Taglialatela et al found that deletion of SMARCAL1, an SNF2 family DNA translocation enzyme, could remodel stalled replication fork and restored its stability, as well as minimize replication stress-induced DNA breaks and chromosomal aberration formation in BRCA1/2 mutated cells.^77^ It has been found that EZH2 locates at a fork of stasis, where it methylates Lys27 on histone 3 (H3K27me3) to mediate the recruitment of MUS81 nuclease.^76^ Inhibition of EZH2 in a mouse BRCA2^−/−^ breast cancer model is associated with acquired PARPi resistance. Thus, loss of the EZH2/MUS81 axis function could promote the resistance of BRCA2-deficient cells to PARPi. These findings suggest that low expression of EZH2/MUS81 predicts PARPi resistance and worse outcomes in BRCA2 mutated tumors.^78^

### Epigenetic modification and restoration of ADPribosylation (PARylation)

Epigenetic modifications may also affect sensitivity to PARPi, leading to acquired resistance to PARPi. Some preclinical studies found that silencing BRCA1 or RAD51C genes by methylation leads to HRR defects (HRD).^79^ The methylation of BRCA1 promoter was first identified in sporadic breast cancer in 1997, and PARPi therapy was subsequently recommended for BRCA1 methylated cancers.^80^^,^^81^ Demethylating agent 5-azacytidine can eliminate PARPi sensitivity in BRCA1-methylated breast cancer cell lines.^79^ Kondrashova reported that the efficacy of rucaparib was demonstrated in PDX models using 12 high-grade serous ovarian carcinoma (HGSOC) patients.^82^ Furthermore, if multiple copies of BRCA1 are present in a tumor, all copies must be methylated to develop a PARPi response, and the loss of only one methylated copy of BRCA1 is sufficient to restore HRR DNA repair and induce PARPi resistance. Response to rucaparib prompted that heterozygous methylation is also associated with PARPi resistance. Loss of methylation in the BRCA1 promoter driven by prior chemotherapy increases the likelihood of acquired resistance to PARPi.^82^ Homozygous RAD51C methylation is also a potential predictive biomarker of sensitivity to PARPi. Collectively, a single non-methylated copy of the gene is sufficient for the development of resistance.^83^

### Others

High mobility group box 3 (HMGB3) is highly expressed in stem cells and cancer cells and is rarely activated in normal adult tissue. This suggests that HMGB3 is a promising therapeutic target.^84^^,^^85^ HMGB3 is associated with radioresistance in cervical cancer,^86^ and tamoxifen resistance in breast cancer.^87^ A recent study showed that targeting HMGB3 consumption by inhibiting ATR/CHK1/p-CHK1 DNA damage signaling pathway sensitizes chemotherapy-resistant ovarian cancer cells to cisplatin.^88^ Ma et al found that HMGB3 was abnormally overexpressed in HGSOC tissues, and the high level of HMGB3 was associated with a shorter OS and a higher risk of resistance to PARPi. PARP1 was identified as a new interaction partner of HMGB3, and the loss of HMGB3 resulted in the trapping of PARP1 at DNA damage sites. Therefore, HMGB3 interaction with PARP1 facilitates its escape from trapping and promotes PARPi resistance.^89^

Signal transducer and activator of transcription 3 (STAT3), known as a transcription factor,^90^ can play a role in promoting tumor by inhibiting anti-tumor immune response.^91^ It has been shown that siRNA-mediated PARP1 or a drug that inhibits PARP1 could lead to increased STAT3 phosphorylation in ovarian cancer cell lines.^92^ Whether PARPi therapy promotes drug resistance by activating STAT3 in ovarian cancer patients has become a question worth exploring. Martincuks et al found that STAT3 activity was significantly enhanced in tumor cells, tumor-associated immune cells, and fibroblasts after PARPi treatment. This increase in activity leads to PARPi resistance and immunosuppression. Elimination of STAT3 may inhibit the growth of PARPi-resistant ovarian tumor cells and restore the sensitivity to PARPi.^93^

Cloning may also play a role in drug resistance. A recent study^94^ used CRISPR/Cas9 technology to obtain TP53 and BRCA1 knockout epithelial cell lines. Seven single-cell clones with acquired resistance after PARPi treatment were obtained. These clones presented increased levels of genomic instability and lower mutational burden with unique transcriptional and mutational profiles compared with PARPi-sensitive cell lines. Clonal analysis showed that acquired PARPi resistance variants were produced by clonal selection. In an image-based drug sensitivity analysis, these clones showed a heterogeneous response pattern based on diverse resistance molecular mechanisms, thus there is an urgent to identify vulnerabilities to the selected agents.^94^

Oxygen levels inside the tumor are uneven, reaching as high as 2% in some areas and less than 0.01% in others. Hypoxia, a property not found in normal cells, could be a target for cancer treatment.^95^ Hypoxia, as a hallmark feature of the tumor microenvironment, could induce resistance to chemotherapy.^96^ Moderate hypoxia (oxygen <2%) promotes the resistance of HR-proficient and/or deficient cancer cells to PARPi in a HIF-independent pathway.^97^ However, some studies have shown that the anoxic conditions with oxygen content less than 0.2% can increase the sensitivity of HR-proficient tumors to PARPi with synthetic lethal effects.^98^ Theoretically, the reduction of ROS-induced DNA damage was responsible for the observed resistance. Remarkably, it has been shown that the hypoxic cytotoxin tirapazamine, targeting hypoxic tumor cells, could enhance the efficacy of PARPi therapy.^97^

To sum up, PARPi has a variety of resistance mechanisms. BRCA1/BRCA2 reverse mutations,^60^ epigenetic modification and restoration of ADPribosylation (PARylation),^82^ restoration of homologous recombination repair^75^ or replication fork protection^76^ could be found in ovarian cancer. Drug efflux,^58^ BRCA1/BRCA2 reverse mutations,^59^ epigenetic modification and restoration of PARylation^79^ were found in breast cancer. However, among those different resistance mechanisms, only HR reverse mutations have been found in patients in clinical trials. Therefore, further research is needed to provide evidence of all the different resistance mechanisms in clinical trials.

## Solutions to PARPi resistance

Overcoming PARPi resistance and identifying predictive biomarkers for PARPi response have been investigated in recent years and will have a great impact in the era of precision medicine.

### Identifying novel predictive biomarkers

Several studies have revealed that RAD51 has the potential to be a novel predictive biomarker.^38^^,^^99^, ^100^, ^101^ The detection of RAD51 foci using immunohistochemistry was associated with the functional status of BRCA1/2 shown in genomic data, as well as the response to DNA damage treatment, supporting the formation of these foci as a clinically useful biomarker.^57^^,^^65^ Down-regulation of RAD51 using shRNA sensitized cancer stem cells (CSCs) to PARPi and inhibited tumor growth in triple-negative breast cancer.^102^ Bermejo et al analyzed the predictive function of RAD51 score and HR defect in breast cancer PDXs, reporting a clear distinction in RAD51 between PARPi-sensitive and -resistant cells. The RAD51 score outperformed gene sequencing in terms of detecting PARPi sensitivity and resistance.^100^^,^^101^ The above studies indicate that RAD51 is a valuable biomarker for predicting PARPi resistance.

**Figure 4:**
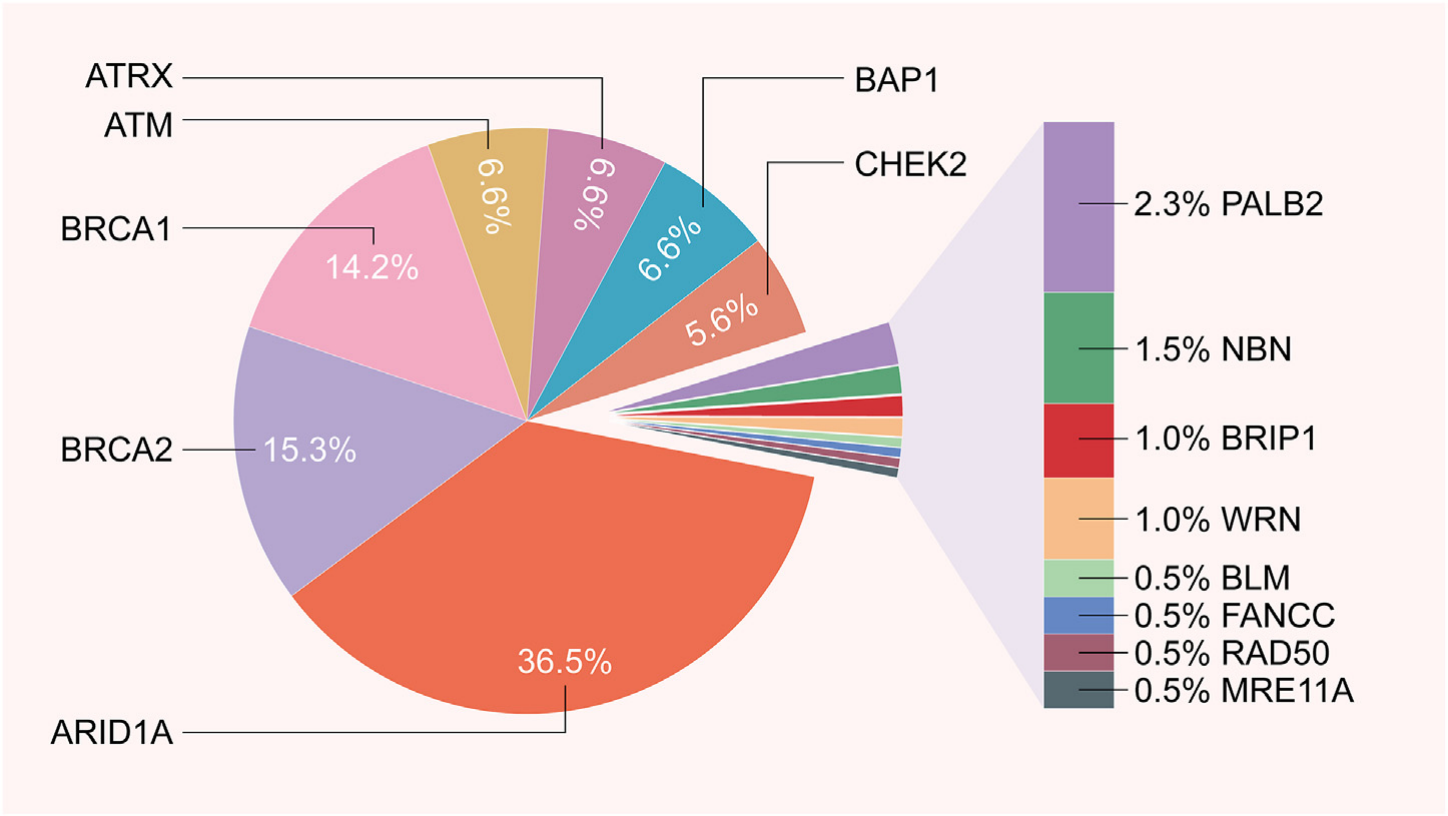
The mutation frequencies of 15 HR-DDR genes. The HR-DDR genes shown here are ARID1A, ATM, ATRX, BAP1, BARD1, BLM, BRCA1/2, BRIP1, CHEK1/2, FANCA/C/D2/E/F/G/L, MRE11A, NBN, PALB2, RAD50, RAD51, RAD51B, and WRN.

The identification of other RAD51-like biomarkers will expand the use of PARPi in more clinical applications and benefit more patients. After reviewing the molecular profiles of 52,426 tumor samples from 21 cancer types, Heeke AL et al identified the frequencies of pathogenic mutations in HR-DDR genes (ARID1A, ATM, ATRX, BAP1, BARD1, BLM, BRCA1/2, BRIP1, CHEK1/2, FANCA/C/D2/E/F/G/L, MRE11A, NBN, PALB2, RAD50, RAD51, RAD51B, and WRN). The frequencies of HR mutations were 15.6% in triple-negative breast cancers, 18.1% in melanoma, 15.4% in pancreatic cancer, and 15.0% in CRC among others. Regarding the pathogenic mutation frequency of the HR pathway genes, ARID1A (7.2%) was the most mutated gene in HR pathway, followed by other 14 genes including BRCA2 (3.0%), BRCA1 (2.8%), ATM (1.3%), ATRX (1.3%), and CHEK2 (1.3%).^103^ The proportion of mutation frequency of these 15 genes is demonstrated in Figure 4. Recently, Ipsen et al identified six candidate genes with the utility of genome-wide CRISPR-Cas9. Multiple knockout populations/clones of each of the six genes were then generated in C4 and/or LNCaP CRPC cells, confirming that the deletion of PARP1, ARH3, YWHAE, or UBR5 caused resistance to olaparib. Drug resistance caused by the knockout of PARP1 and ARH3 may be related to decreased autophagy, but further research is needed.^104^ Peng et al found that the deubiquitylating enzyme USP15 regulates homologous recombination repair and attenuates the response to PARPi in cancer cells. USP15 is recruited to DSBs and deubiquitinates the BRCT domain of BRCA1-associated RING domain protein 1 (BARD1) to promote BRCA1/BARD1 retention and function in DSBs. Consequently, overexpression of USP15 will largely contribute to the occurrence of drug resistance.^105^ In the near future, advanced sequencing techniques can be used to select PARPi-sensitive patients and reduce the occurrence of drug resistance.

### Combination with other targeted therapies

Numerous studies have investigated how to overcome PARPi resistance pathways based on molecular mechanisms. Some studies have made breakthrough progress in drug combination. The combination of PARPi and other targeted drugs can make patients re-sensitive to PARPi, or improve the efficacy to achieve synthetic lethality.

First, unexpected synergistic cytotoxic effects were achieved by the addition of mitogen-activated protein kinase (MAPK) kinase (MEK) inhibitors to PARPi. Sun et al found that RAS-MEK-ERK signaling in cancer cells is up-regulated in multiple RAS-mutated tumor models *in vivo* and *in vitro*.^106^ RAS pathways are also activated in PARPi-resistant cells. The efficacy of PARPi combined with MEK inhibitor was independent of the mutational status of BRCA1/2 and TP53, suggesting that synergies may be expandable.^106^ Moreover, MEK inhibitors can reverse resistance to PARPi therapy.^107^ These results indicate that the addition of MEK inhibitor to PARPi not only improves the efficacy but also overcomes drug resistance.

Second, from the perspective of the structure and mechanism of PARPi action, the resistance to PARPi can be reversed by inhibiting P protein or exposing ssDNA gap or dePARylation. The reduction in intracellular P-glycoprotein concentration by efflux of PARPi leads to resistance to PARPi. This re-sensitization to PARPi can be found by combination therapy with the p-glycoprotein inhibitor tariquidar.^58^ In addition, the ssDNA gap is also an important therapeutic target for drug resistance. When conducting functional genetic screening in PARPi-resistant cells and organoids through 53BP1 deletion, Paes Dias et al found that deletion of nuclear DNA ligase III (LIG3) could enhance PARPi toxicity in BRCA1-deficient cells. It was found that LIG3 deletion promoted the formation of MRE11-mediated post-replication ssDNA gaps in BRCA1-deficient and BRCA1/53BP1 double-deficient cells, and then increase the exposure to PARPi and could reverse PARPi resistance in BRCA1/53BP1 double-deficient cells.^108^ JH-RE-06 has a potentially toxic effect on PARPi-resistant BRCA1 mutant cells by inhibiting the repair of ssDNA gaps. Furthermore, it displays additive toxicity with crosslinking agents or PARPi.^109^ With the utility of COH34, a small molecule targeting poly (ADP-ribose) glycohydrolase (PARG), Chen et al identified its novel role of dePARylation in DNA repair. COH34 binds to the CAT domain of PARG, a major dePARylation enzyme, and then could prolong PARylation at the site of DNA damage and trap DNA repair factors. COH34 was shown not only to have a lethal effect on cancer cells with DNA repair deficiency but also to effectively kill PARPi-resistant cancer cells.^110^ Prexasertib, a checkpoint kinase 1 (CHK1) inhibitor, can lead to replication catastrophe in PARPi-resistant HGSOC PDX models and cell lines. The combination of olaparib with prexasertib could not only significantly slow the tumor growth in olaparib-resistant models, but also further enhance the magnitude and persistence of response in olaparib-sensitive models. Moreover, prexasertib reverses restored HR and replication fork stability, acting synergistically with PARPi.^111^

Third, combination with other targeted agents could enhance the anti-tumor effect of PARPi. Two recent studies have shown that pyruvate kinase M2 (PKM2) inhibitor and ALK kinase inhibitor ceritinib could induce DNA damage in ovarian cancer cells and improve the response to olaparib, respectively.^112^^,^^113^ Gabbasov et al revealed heat shock protein 90 (HSP90) regulates the maturation and stability of a key protein required for DDR. It also indicates that the addition of ganetespib, a unique small molecule HSP90 inhibitor, could effectively disrupt key DDR pathway proteins and make ovarian cancer cells without “BRCAness” respond to talazoparib.^113^^,^^114^ In addition, combination therapy also works in breast cancer. Wang et al demonstrated that the limited response of BRCA1-deficient breast tumors to PARPi is mainly due to M2-like pro-tumor macrophages, which inhibits CD8^+^ T cells in the immune system and impedes PARPi-triggered tumor cell DNA damage as well. The addition of exogenous STING agonists can transform tumor-associated macrophages (TAMs) from M2-like into an M1-like anti-tumor state. Moreover, systemic administration of STING agonist combined with PARPi enhances anti-tumor immunity regardless of STING expression in tumor cells and has shown significant therapeutic effect in BRCA1-deficient breast cancer mouse models.^115^ Li et al found that enzalutamide, an inhibitor of androgen receptor, inhibits the expression of several genes associated with HR in CRPC cell lines, even though it is generally ineffective in CRPC patients. Therefore, the combination of olaparib with enzalutamide could promote cell death in CRPC cells.^116^ Blocking nucleotide salvage factor DNPH1 combined with the treatment with hmdU (5-hydroxymethyl-deoxyuridine) could overcome the resistance of PARPi in BRCA1-deficient cells. Targeting DNPH1 provides a promising strategy for the hypersensitization of BRCA-deficient cancers to PARPi therapy.^117^ In addition, combining PARPi with molecules that target cell cycle checkpoints is also a synergistic approach. The DNA replication stress checkpoint (ATR-CHK1-WEE1) showed promising results among cell cycle checkpoints.^118^ When combined with olaparib, the WEE1 inhibitor provides a unique therapeutic strategy to overcome PARPi resistance by targeting replication stress response.^119^ Kim et al found that the acquired resistance to PARPi was often accompanied by increased ATR-CHK1 activity and ATR inhibitor (ATRi) sensitivity. In a clinically relevant acquired PARPi-resistant PDXs model, a significant increase in survival rate was observed with a durable complete therapeutic response to the combination of PARPi and ATRi.^120^ More robust clinical trial evidence in the future is needed to select the optimal treatment strategy for patients.

### The combination of radiotherapy and immunotherapy

Radiation causes DNA damage in cells, including DSBs, SSBs, and interstrand cross-linking, which disrupts DNA replication and the following transcription and ultimately leads to cell death.^121^ PARP1 inhibitor could enhance ionizing radiation (IR)-induced cytotoxicity by inhibiting NF-κB activation.^122^ Also, PARP2 depletion demonstrated higher sensitivity to the cell-killing effects of IR *in vitro*.^123^ Preliminary data showed that PARPi or PARP depletion has a radiosensitization effect to enhance the efficacy of radiotherapy in ovarian cancer, breast cancer, cholangiocarcinoma, and soft tissue sarcoma.^124^, ^125^, ^126^, ^127^ The addition of DNA methyltransferase inhibitors (DNMTis) to PARPi could further improve the response in NSCLC cells to IR *in vitro* and *in vivo*.^126^ Since nitric oxide enhances HRD by inhibiting BRCA1 expression under oxidative stress, nitric oxide-donor combined with PARPi provides a new approach for sensitization to IR.^128^ These preclinical studies suggested a synergistic effect when combining radiation with PARPi therapy, indicating promising investigations in clinical trials. Several studies have explored how to maximize the benefit of combination therapy *in vitro*. The low dosage of olaparib is associated with higher sensitivity to the combination therapy, particularly in cells with homologous recombination-impaired status.^129^ In HR-proficient tumor types, p53 status was a candidate predictive biomarker, which could be used to select patients who might benefit more from the combination therapy.^130^

Immuno-oncology (IO) therapy activates the ability of the immune system to eliminate cancer cells rather than targeting the tumor directly.^131^ PARPi could transform “cold” tumors into “hot” tumors to improve the response to immunotherapy. PARPi not only increases tumor mutation burden to generate more neoantigens by inhibiting DNA damage repair, but it also induces the expression of PD-L1.^132^ The rationale for the combination of PARPi and immunotherapy has led to numerous subsequent clinical trials.^133^ Pembrolizumab, a PD-1 inhibitor that can block the interaction between PD-1 and its ligands PD-L1/PD-L2, received FDA approval for TNBC in neoadjuvant and metastatic settings. The addition of pembrolizumab to niraparib showed promising anti-tumor activity in advanced TNBC,^134^ refractory platinum-resistant ovarian cancer,^135^ and mCRPC.^136^ There are ongoing studies on the combination of PARPi and anti-PD1/PD-L1 agents in patients with advanced solid cancers.^137^^,^^138^ Encouraging results from these pilot studies revealed the synergistic effect of the combination, warranting further investigation. Also, it should be noted that a subset of patients are not responders, suggesting it is urgent to identify the predictive biomarkers to select patients for combination therapy. Based on the retrospective exploratory research in TOPACIO trial,^135^ potential predictive biomarkers for the efficacy of the niraparib/pembrolizumab combination are mutational signature 3 and interferon signaling in the CD8^+^ T cells in the tumor microenvironment, in addition to HR or BRCA mutation status.^139^ Therefore, predictive biomarkers should be the primary focus of future studies to prospectively identify the ones who would benefit from combination therapy.

## Conclusion

Despite the great progress in using PARPi in clinical treatment for HR-deficient tumors, the emerging drug resistance has dampened the initial enthusiasm. Deeply understanding the mechanisms of resistance to PARPi and developing novel combination therapies to overcome drug resistance are essential to optimize the use of PARPi and benefit more patients.

## Conflict of interests

The authors declare no conflict of interests.
